# Spatial patterns of white matter hyperintensities associated with Alzheimer’s disease risk factors in a cognitively healthy middle-aged cohort

**DOI:** 10.1186/s13195-018-0460-1

**Published:** 2019-01-24

**Authors:** Gemma Salvadó, Anna Brugulat-Serrat, Carole H. Sudre, Oriol Grau-Rivera, Marc Suárez-Calvet, Carles Falcon, Karine Fauria, M. Jorge Cardoso, Frederik Barkhof, José Luis Molinuevo, Juan Domingo Gispert, for the ALFA Study, Jordi Camí, Albina Polo, Cristina Mustata, Laia Tenas, Paula Marne, Xavi Gotsens, Tania Menchón, Anna Soteras, Laura Hernandez, Ruth Dominguez, Sandra Prades, Raffaele Cacciaglia, Grégory Operto, Stavros Skouras, Gonzalo Sánchez, Carolina Minguillón, Gema Huesa, Marc Vilanova, Sabrina Segundo, Jordi Huget

**Affiliations:** 1 Barcelonaβeta Brain Research Center, Pasqual Maragall Foundation, Wellington 30, 08005 Barcelona, Spain; 2 Engineering and Imaging Sciences, King’s College London, London, UK; 3 Dementia Research Centre, University College London, London, UK; 4 Centre for Medical Imaging Computing, Faculty of Engineering, University College London, London, UK; 5 Centro de Investigación Biomédica en Red de Bioingeniería, Biomateriales y Nanomedicina (CIBER-BBN), Madrid, Spain; 6 Brain Repair and Rehabilitation, University College London, London, UK; 7 Radiology & Nuclear Medicine, VU University Medical Centre, Amsterdam, Netherlands; 8 Centro de Investigación Biomédica en Red de Fragilidad y Envejecimiento Saludable (CIBERFES), Madrid, Spain; 9 Universitat Pompeu Fabra, Barcelona, Spain

**Keywords:** Vascular, Lesions, Aging, Brain, Prevention

## Abstract

**Background:**

White matter hyperintensities (WMH) of presumed vascular origin have been associated with an increased risk of Alzheimer’s disease (AD). This study aims to describe the patterns of WMH associated with dementia risk estimates and individual risk factors in a cohort of middle-aged/late middle-aged individuals (mean 58 (interquartile range 51–64) years old).

**Methods:**

Magnetic resonance imaging and AD risk factors were collected from 575 cognitively unimpaired participants. WMH load was automatically calculated in each brain lobe and in four equidistant layers from the ventricular surface to the cortical interface. Global volumes and regional patterns of WMH load were analyzed as a function of the Cardiovascular Risk Factors, Aging and Incidence of Dementia (CAIDE) dementia risk score, as well as family history of AD and *Apolipoprotein E* (*APOE*) genotype. Additional analyses were performed after correcting for the effect of age and hypertension.

**Results:**

The studied cohort showed very low WMH burden (median 1.94 cm^3^) and 20-year dementia risk estimates (median 1.47 %). Even so, higher CAIDE scores were significantly associated with increased global WMH load. The main drivers of this association were age and hypertension, with hypercholesterolemia and body mass index also displaying a minor, albeit significant, influence. Regionally, CAIDE scores were positively associated with WMH in anterior areas, mostly in the frontal lobe. Age and hypertension showed significant association with WMH in almost all regions analyzed. The *APOE-ε2* allele showed a protective effect over global WMH with a pattern that comprised juxtacortical temporo-occipital and fronto-parietal deep white matter regions. Participants with maternal family history of AD had higher WMH load than those without, especially in temporal and occipital lobes.

**Conclusions:**

WMH load is associated with AD risk factors even in cognitively unimpaired subjects with very low WMH burden and dementia risk estimates. Our results suggest that tight control of modifiable risk factors in middle-age/late middle-age could have a significant impact on late-life dementia.

**Electronic supplementary material:**

The online version of this article (10.1186/s13195-018-0460-1) contains supplementary material, which is available to authorized users.

## Introduction

White matter hyperintensities (WMH) are frequently observed on T2-weighted magnetic resonance imaging (MRI) sequences of healthy middle-aged and elderly individuals [1–3]. They are thought to be associated with axonal loss and demyelination due to chronic ischemia, and therefore are considered as surrogate markers of cerebral small vessel disease, although their histopathological substrate might be heterogeneous [4, 5]. Multiple risk factors of WMH are shared with Alzheimer’s disease (AD) such as ageing, hypertension, hypercholesterolemia, and diabetes [6–10]. Moreover, WMH increase the risk of cognitive decline and AD, contributing to its progression and severity [11–14]. As the therapeutic efforts in the AD field progressively shift toward its prevention, a better characterization of potentially modifiable contributors to the burden of the disease is of the utmost importance [3, 15].

Regarding hereditary risk factors for AD, the *ε4* allele of the *apolipoprotein E* (*APOE*) gene, the main genetic risk factor for sporadic AD [16], has also been associated with increased WMH load [17–23]. However, this association remains controversial [10, 24–26], possibly due to heterogeneity in the methodological approaches. On the other hand, fewer studies have analyzed the association of AD family history and WMH load, again with conflicting results. While some could not detect any relationship with WMH [27, 28], others reported an increased WMH load in participants with maternal family history or with both parents affected [29].

The CAIDE (Cardiovascular Risk Factors, Aging, and Incidence of Dementia) dementia risk score has been developed to predict the risk of dementia in 20 years among middle-aged individuals [6]. This scale assigns scores to participants on their characteristics, taking into account some of the most important risk factors for dementia (age, education, sex, systolic blood pressure, body mass index (BMI), total cholesterol, physical activity, and *APOE* status). Once a participant has their CAIDE dementia risk score assigned, their risk of dementia after 20 years can be derived.

Associations between CAIDE scores and WMH have been previously reported. One study found that middle-aged participants with a CAIDE score above nine had increased volumes of WMH 20 years later [30]. Recently, those increments were reported to be only present in deep white matter (DWM) [31]. However, only one study has focused this analysis on cognitively normal middle-aged subjects [32] and found that individuals at high risk (CAIDE > 9) showed higher WMH burden, as measured with the semiquantitative Fazekas visual scale, and that WMH mediated the relation between CAIDE with executive function as well as visual perception and construction abilities.

Until recently, the literature has mainly concentrated on global cerebral WMH measures. Lately, more studies have started to focus on investigating the relevance of the topographical distribution of WMH [27, 33]. In those studies, specific spatial patterns of WMH were related to relevant vascular [33] and AD risk factors [18]. Moreover, strategically located WMH might have an impact on cognition [34, 35] and increase the risk of developing AD [36]. Most of those studies, however, were carried out with relatively old and/or cognitively impaired participants, often presenting with several comorbidities.

In the present study, we sought to investigate the association between global and regional WMH burden and CAIDE dementia risk score in a cohort of middle aged/late middle-aged cognitively healthy participants enriched for heritable AD risk factors. The fact that our participants are younger and cognitively healthier than the ones in previous studies allow us to better understand the role of modifiable and nonmodifiable risk factors of dementia into WMH burden and distribution, without the mixturing effect of other comorbidities. Therefore, global and regional associations between WMH and each individual risk factor included in the CAIDE scale, including the *APOE* genotype, as well as family history of AD have been studied.

## Methods

### Participants

Participants in this study are part of a wider research platform: the ALFA cohort (for ALzheimer and FAmilies). With the aim of tracking the evolution of the AD continuum in asymptomatic individuals, the ALFA cohort is composed of 2743 cognitively normal participants, many of them adult children of patients with AD, aged between 45 and 75 years. The ALFA study protocol was approved by the Independent Ethics Committee Parc de Salut Mar Barcelona (and registered at Clinicaltrials.gov, NCT01835717). For a full detailed description of the cohort see Molinuevo et al. [37]. In brief, participants had a Clinical Dementia Rating (CDR) score [38] equal to 0 and scored within the established cut-offs for the neuropsychological battery that included the Mini-Mental State Examination (MMSE) ≥ 26, Memory Impairment Screen (MIS) ≥6 [39, 40], Time-Orientation subtest of the Barcelona Test II (TO-BTII) ≥68 [41], and semantic fluency (animals, SF) ≥12 [42, 43]. Exclusion criteria for these participants included major psychiatric disorders or other diseases that could affect cognition, neurological disorders, brain injury that could affect cognition, or family history of AD with suspected autosomal dominant pattern.

A subgroup of 608 ALFA participants without MRI contraindications was selected to participate in the present study according to their *APOE* genotype, preferentially including *APOE-ε4* and *APOE-ε2* carriers [44]. The rest of the participants were selected to try and match the previous subjects by age and sex. The MRI study protocol, registered at Clinicaltrials.gov (NCT02198586), has been conducted in accordance with the directives of the Spanish Law 14/2007, of 3 July, on Biomedical Research (Ley 14/2007 de Investigación Biomédica). All participants accepted the study procedures by signing an informed consent form.

From the 608 participants invited to participate in this study, 595 agreed to undertake MRI scans and 575 provided valid MRIs. The most important issue for valid MRI acquisition was claustrophobia (*n* = 16 drop-outs), followed by three participants of incompatible physical size or shape that precluded lying in the scanner, and an image artifact caused by irremovable metallic earrings (*n* = 1). Finally, we also have to remove the scans of 14 participants from this study due to the presence of incidental findings [2], problems in motion artifacts, or segmentation problems that prevented us correctly segmenting WMH. Therefore, a total of 561 images were available for subsequent analyses.

### Sociodemographic, anthropometric, lifestyle, and clinical factors

Basic sociodemographic and clinical data were registered either during the clinical interview or through on-line, self-administered questionnaires. All participants were asked about their family and personal medical history, and medication use was recorded. Participants were considered to be ‘hypertensive’ if at least one of the following conditions was met: 1) participant self-reported diagnosis; 2) current use of medication; 3) measured systolic blood pressure above 140 mmHg. Analysis of global and regional WMH load for each of these conditions is shown in Additional file 1. Note that, only condition 3 was used for the derivation of the CAIDE score (see the CAIDE dementia risk score derivation section below). ‘Hypercholesterolemia’ was categorized as present if at least one of the first two aforementioned conditions for hypertension were met.

BMI was derived from the height and weight measured at the time of the interview. Physical activity was measured using the Spanish short version of the Minnesota Leisure Time Physical Activity Questionnaire [45], and participants were split into two categories: ‘active’ or ‘inactive’. A participant was considered to be active if he/she did more than 150 min of moderate exercise or 75 min of vigorous exercise per week as recommended by current guidelines.

### CAIDE dementia risk score derivation

The previously mentioned factors were used to derive the probability of dementia in 20 years using the CAIDE dementia risk score [6], as explained previously [17]. In brief, CAIDE takes into account the age, education, sex, systolic blood pressure, BMI, total cholesterol, and physical activity in the first model (CAIDE-I), and also *APOE* status in the second model (CAIDE-II). Only the first model is reported in this study due to a high similarity in the results of both models. In addition, CAIDE-II only considers whether an individual carries a copy of the *ε4* allele, whereas in our study we sought differences among all combinations of alleles (see the *APOE* genotyping section below).

For the calculation of CAIDE dementia risk scores, each participant was assigned some points or scores depending on the aforesaid characteristics. Additional file 1 Table S1 summarizes the risk factors taken into account and the correspondent scores assigned to the participant for those characteristics. Once the total CAIDE dementia risk score is calculated, a percentage risk of dementia 20 years later can be derived using a nonlinear expression that can be found in the reference paper [6].

All the necessary information to derive CAIDE dementia risk scores was registered in the ALFA cohort with the exception of total cholesterol in blood. To take into account this measure, we assigned 2 points (as with participants with > 6.5 mmol/l in the original CAIDE derivation) to those participants who reported to be diagnosed with hypercholesterolemia or to be taking medication to control it. Regarding hypertension status, we used the same criterion as in the original paper, classifying as hypertensive those participants with systolic blood pressure above 140 mmHg. All the rest of the CAIDE scores were assigned as in the original work.

### *APOE* genotyping

Total DNA was obtained from the blood cellular fraction by proteinase K digestion followed by alcohol precipitation. Using the following primers (*APOE-F 5′ -TTGAAGGCCTA CAAATCGGAACTG-3′* and *APOE-R 5′ -CCGGCTGCCCAT CTCCTCCATCCG-3′*) samples were genotyped for two single nucleotide polymorphisms (SNPs), rs429358 and rs7412, determining the possible *APOE* alleles: *ε1*, rs429358 (C) + rs7412 (T); *ε2*, rs429358 (T) + rs7412 (T); *ε3*, rs429358 (T) + rs7412 (C); and *ε4*, rs429358 (C) + rs7412 (C). All allele combinations were considered as separate categories for subsequent statistical analyses.

### Family history of AD

Family history of AD was recorded as previously reported [37]. In brief, family history was divided into four possible groups: ‘maternal’, ‘paternal’, ‘both parents’, and ‘no AD family history’. This classification was only considered positive if the antecedent relative was younger than 75 years at the time AD was diagnosed.

### MRI acquisition

MRIs were acquired on a 3.0-T scanner (GE Discovery MR750 W 3 T). The same protocol, which included one T1- and three T2-weighted sequences, was performed on all participants. The T1-weighted sequence had an isotropic voxel size of 1 mm^3^ with a matrix size of 256 × 256 × 160 (TR/TE/TI = 8.0/3.7/450 ms, NSA = 1, flip angle = 8°). T2 and T2*-weighted sequences, with a voxel size of 1 × 1 × 3 mm, were as follows: fluid attenuation inversion recovery (FLAIR: TR/TE/TI = 11,000/90/2600 ms, flip angle = 160°), fast spin echo (TR/TE = 5000/85 ms, flip angle = 110°), and gradient echo (GRE: TR/TE = 1300/23 ms, flip angle = 15°). All scans were visually assessed for quality and incidental findings by a trained neuroradiologist [2].

### WMH visual assessment

All MRIs were visually assessed by a trained neuroradiologist who was blinded to the *APOE* genotype of the participants. All images were rated using modifications of the Fazekas Scale [46], which separately categorizes the severity of deep and periventricular lesions on a scale from 0 to 3 (0, none or a single punctate WHM lesion; 1, multiple punctate lesions; 2, beginning confluency of lesions (bridging); and 3, large confluent lesions).

### WMH segmentation and quantification

WMH were automatically segmented using a Bayesian algorithm [47]. In brief, T1-weighted, T2-weighted, and T2-FLAIR images are rigidly coregistered using the NiftyReg package [48]. The data are then modeled as a multivariate Gaussian mixture model that simultaneously accounts for healthy tissue and unexpected observations and is constrained by participant-specific statistical tissue priors derived from the Geodesic Information Flows (GIF) algorithm [49].

The number of required Gaussian components is dynamically determined at a patient level to ensure a balance between model fit and complexity using the Bayesian Inference Criterion. Once the model has converged, a postprocessing step is applied to extract probability maps of candidate lesion voxels that are then further corrected for spurious false positive detection using the output of the parcellation algorithm to avoid regions prone to artefacts. Volumetric measurements are derived as the sum of this probability map over a region of interest.

This method was applied only for supratentorial regions, therefore excluding cerebellar and brainstem areas. We also calculated the total intracranial volume (TIV) for normalization purposes. This measure was derived automatically using a previously described method [49], and it included total brain volume comprising also ventricles and cerebrospinal fluid (CSF) .

**Fig. 1:**
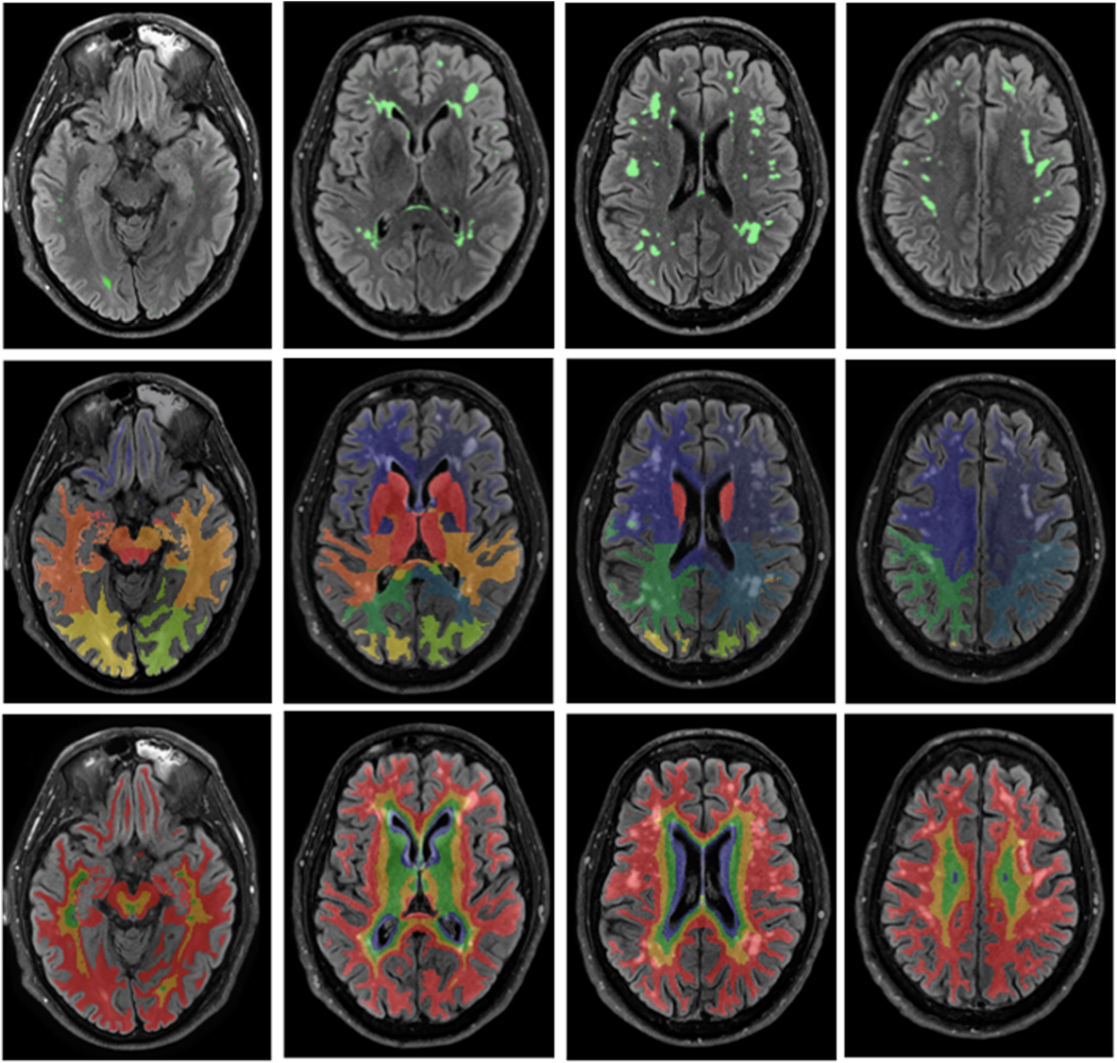
Example of the brain segmentation for one participant. Different axial slices of the same participant are shown in each column. The first row shows WMH lesion segmentation in green. In the second row, lobar segmentation is shown. Finally, the last row shows the four layers in which each lobe was segmented. Of these layers, the most internal represents periventricular areas, there are two layers of DWM and finally a juxtacortical layer which is the most external

To depict regional results, we used a bullseye representation [50] (excluding infratentorial regions). Every sector of the bullseye represents one lobar white matter segment obtained based on the cortical parcellation output from the GIF algorithm. Another unique region was the basal ganglia (including internal capsule and the thalamus). The concentric rings in the bullseye plot are defined by dividing the area between the ventricular surface and the cortical sheet into four equidistant layers. The interior layer in the plot represents the most periventricular area and the most external layer corresponds to the juxtacortical regions. The final representation is formed of 36 regions that are composed of nine-lobar segmentation with four layers each. Figure 1 shows an example of the segmentation for one participant.

### Statistical analyses

We sought to assess the association between global and regional WMH load with CAIDE-I scores and with each of the individual risk factors included in the scale, namely age, hypertension, hypercholesterolemia, BMI, sex, education, and physical exercise. We also assessed the association with *APOE* genotype and family history of AD. All the analyses had the WMH percentage of the TIV as an outcome variable to account for total brain size. The distribution of the main dependent variables (global and regional WMH load) was significantly different from normal (Additional file 1: Figure S1A), as well as after log-transformation. For this reason, we used nonparametric statistics for all the analyses.

First, we tested the cross-correlation between CAIDE percentage risk of dementia and its individual risk factors. To do so, we used Spearman’s rank test (continuous vs. continuous), Mann-Whitney *U* test (continuous vs. dichotomous), and χ^2^ test (dichotomous vs. dichotomous). The effect size for each of these tests were Spearman’s rho, Cohen’s *d*, and φ, respectively.

Then we sought associations between WMH load and independent variables, also using nonparametric tests: Spearman’s rank correlation for continuous variables and Mann-Whitney *U* test for dichotomous variables. To test the associations of CAIDE we used the percentage risk of having dementia as a predictor.

We also analyzed the association between global and regional WMH load against *APOE* and family history of AD. As these variables had more than two groups all comparisons were performed against a reference group. In *APOE* analysis we compared all the genotypic groups to *APOE-ε3/ε3* participants (except for *ε2* homozygotes due to a low number). When comparing family history of AD, those participants without family history of AD were set as reference group.

A modified bootstrap method was used to calculate *p* values. We randomly assigned the outcome variables to the original predictors 10,000 times, without resampling, and for each re-assignation we calculated the Spearman’s rho or *z* statistic. Then *p* values were estimated by calculating the number of times the statistic was higher/lower (two-sided test) than the statistic calculated with the original data, and then dividing by the number of permutations done. The threshold for statistical significance was set to *p* < 0.05 false discovery ratio (FDR)-corrected. The effect size shown in the figures is either Spearman’s rho or Cohen’s *d*, depending on the nature of the variable (continuous or categorical).

Three different statistical models were used in all the studied comparisons. In the first model, no covariates were included to show the direct associations between WMH and the predictors. In the second model, we corrected for the effect of age and, in the last one, for hypertension status, but not age. The reason for correcting for these two factors was two-fold. First, they were expected to be the main drivers of the association of CAIDE and WMH and, second, some groups created for dichotomous variables (e.g., women/men) differed significantly on one of these two main risks (see Additional file 1: Tables S1–S6). Therefore, correcting by these two important confounders is expected to provide more comparable results of the analysis of risk factors in groups unbalanced for them. Finally, we only report here the groups showing significant results for *APOE* and family history of AD analyses.

## Results

The sociodemographic and clinical information of the 561 participants is presented in Table 1. CAIDE dementia risk score was low [6, 30–32] due to the low prevalence of hypercholesterolemia, BMI higher than 30 kg/m^2^, the high number of active subjects, and the high education of the participants [37]. Due to the recruitment strategy of the MRI study, there was a high proportion of *APOE-ε2* and *APOE-ε4* participants and, more specifically, *APOE-ε4* homozygotes. Likewise, a high proportion of participants also presented a family history of AD, more often from the maternal side.

**Table 1:** Sociodemographic and clinical characteristics of ALFA participants

Sociodemographic and clinical variables (*n* = 561)	Mean / n	Interquartile range / %
CAIDE-I risk (%)	1.47	0.67–3.22
CAIDE-I score (0–15)	6	4–8
Age (years)	58	51–64
Hypertension, *n* (%)	147	26.2
Hypercholesterolemia, *n* (%)	171	30.5
BMI (kg/m^2^)	26.4	24.0–29.4
Sex (male), *n* (%)	219	38.9
Education (years)	14	11–17
Physical exercise, *n* (%)		
Active	368	65.6
Not active	148	26.4
Not available	45	8.0
*APOE* genotype, *n* (%)		
*ε4/ε4*	71	12.7
*ε4/ε3*	170	30.3
*ε3/ε3*	159	28.3
*ε2/ε4*	45	8.0
*ε2/ε3*	109	19.4
*ε2/ε2*	7	1.2
Family history of AD, *n* (%)		
Maternal	190	33.9
Paternal	91	16.2
From both	16	2.9
None	245	43.7
Not available	19	3.4
Fazekas Scale, *n* (%)		
Score 0	269	48.0
Score 1	247	44.0
Score 2	43	7.7
Score 3	2	0.4
Total WMH load (cm^3^)	1.94	1.13–3.69
Total WMH load/TIV (%)	0.14	0.08–0.26
MMSE	29	28–30
Time difference between visit and MRI (days)	307	251–366

Values are expressed as median (interquartile range) unless otherwise indicated

*AD* Alzheimer’s disease, *APOE* apolipoprotein E, *BMI* body mass index, *CAIDE* Cardiovascular Risk Factors, Aging, and Incidence of Dementia, *MMSE* Mini-Mental State Examination, *MRI* magnetic resonance imaging, *TIV* total intracranial volume, *WMH* white matter hyperintensities

Correlations among CAIDE and its individual components are shown in Additional file 1: Figure S2. CAIDE showed a high correlation with all risk factors included in its calculation, as was expected. Among individual risk factors, age was significantly and positively associated with hypertension, hypercholesterolemia, and BMI, and negatively to education. Male sex was significantly associated with higher prevalence of hypertension as well as with higher BMI and education.

WMH burden was low [27, 33, 36] as, on average, it only covered a volume of 1.94 cm^3^ corresponding to 0.14% of the TIV. Only 8% of our cohort reached pathological levels of WMH with the Fazekas scale (≥ 2 score on this age range) [46]. Regarding the spatial distribution of WMH, the most affected regions were periventricular areas and the occipital lobe (Additional file 1: Figure S1C).

### Global analysis

The CAIDE percentage risk of dementia was significantly associated with global WMH (Model 1, effect size 0.11, 95% confidence interval (CI) 0.03–0.19, *p* = 0.017; Table 2). However, this association became nonsignificant after adjusting for the effect of age or hypertension status (Models 2 and 3, respectively).

**Table 2:** Associations between global WMH and AD and WMH risk factors

	Model 1 (Direct)		Model 2 (Age-corrected)		Model 3 (Hypertension-corrected)	
	Effect size (95% CI)	*p*	Effect size (95% CI)	*p*	Effect size (95% CI)	*p*
CAIDE-I	**0.11 (0.03 to 0.19)**	**0.017**	0.01 (−0.07 to 0.09)	0.423	0.01 (−0.08 to 0.09)	0.444
Age	**0.22 (0.13 to 0.29)**	**< 0.001**	–	–	**0.18 (0.09 to 0.26)**	**< 0.001**
Hypertension	**0.14 (0.05 to 0.22)**	**< 0.001**	**0.09 (0.00 to 0.17)**	**0.017**	–	–
Hypercholesterolemia	**0.09 (0.00 to 0.17)**	**0.042**	0.05 (−0.04 to 0.14)	0.207	0.07 (−0.02 to 0.15)	0.051
BMI	0.08 (−0.00 to 0.16)	0.053	0.06 (−0.02 to 0.14)	0.207	0.03 (−0.05 to 0.11)	0.249
Sex (Men)	−0.01 (−0.09 to 0.08)	0.411	−0.01 (−0.10 to 0.07)	0.423	−0.03 (−0.11 to 0.05)	0.246
Education	−0.04 (−0.13 to 0.04)	0.192	−0.01 (−0.09 to 0.08)	0.423	−0.02 (−0.11 to 0.06)	0.313
Physical exercise	−0.04 (−0.13 to 0.04)	0.192	−0.05 (−0.14 to 0.03)	0.207	−0.02 (−0.10 to 0.07)	0.352
Maternal family history of AD*	0.12 (−0.02 to 0.17)	0.077	**0.12 (0.03 to 0.21)**	**0.024**	**0.08 (−0.01 to 0.18)**	**0.040**
APOE-ε2/ε3*	**−0.13 (−0.24 to -0.01)**	**0.042**	−0.08 (−0.20 to 0.04)	0.2077	−0.10 (−0.21 to 0.02)	0.054

The three models had WMH/TIV (%) as an outcome variable; no covariates were used in Model 1; age effect was corrected for in Model 2 and hypertension was accounted for in Model 3

Significant results are shown in bold (*p* < 0.05, false discovery ratio-corrected)

Participants without a family history of AD and *APOE-ε3* homozygotes were the reference group for group comparisons

*AD* Alzheimer’s disease, *APOE* apolipoprotein E, *BMI* body mass index, *CAIDE* Cardiovascular Risk Factors, Aging, and Incidence of Dementia, *CI* confidence interval, *TIV* total intracranial volume, *WMH* white matter hyperintensities

*All other pairwise group comparisons showed no significant associations and are not shown in the table

In Model 1 (without covariates) the main drivers of the association between CAIDE percentage risk of dementia and global WMH load were age (effect size 0.22, 95% CI 0.13–0.29, *p* < 0.001) and hypertension (effect size 0.14, 95% CI 0.05–0.22, *p* < 0.001). For the hypertension assessment, the condition was more highly correlated with global WMH burden with the use of medication to control hypertension (Additional file 1: Table S2). Hypercholesterolemia also displayed a significant effect (effect size 0.09, 95% CI 0.00–0.17, *p* = 0.042) and BMI showed a trend to significance (effect size 0.08, 95% CI −0.00 to 0.16, *p* = 0.053). In this direct model we also found lower WMH burden in *APOE-ε2/ε3* participants than in the reference *APOE-ε3/ε3* group (effect size −0.13, 95% CI −0.24 to −0.01, *p* = 0.042). However, the *APOE-ε2/ε3* group was significantly younger than the reference group (56 vs 60 years old; *p* < 0.001, Additional file 1: Table S7) and, when corrected by age (Model 2), this comparison did not achieve significance. The rest of the comparisons between groups of *APOE* or family history of AD showed nonsignificant differences on WMH load.

In Model 2 (adjusted by age), of the CAIDE risk factors only hypertension remained significantly associated with global WHM (effect size 0.09, 95% CI 0.00–0.17, *p* = 0.017), even though hypertensive and nonhypertensive participants were significantly different in age (62 vs 56 years old; *p* < 0.001, Additional file 1: Table S3). In this model, we also found a significant increase in global WMH load in those subjects with a maternal family history of AD (effect size 0.12, 95% CI 0.03–0.21, *p* = 0.024). This group also showed differences in age with respect to the reference group. Participants with a family history of AD were significantly younger than those without (55 vs 60 years old; *p* < 0.001, Additional file 1: Table S8).

Finally, in Model 3 (adjusted by hypertension), only age from the CAIDE variables remained significantly associated with global WMH (effect size 0.18, 95% CI 0.09–0.26, *p* < 0.001). Maternal family history of AD was also significant in Model 3 (effect size 0.08, 95% CI −0.01 to 0.18, *p* = 0.040).

### Regional analysis

**Fig. 2:**
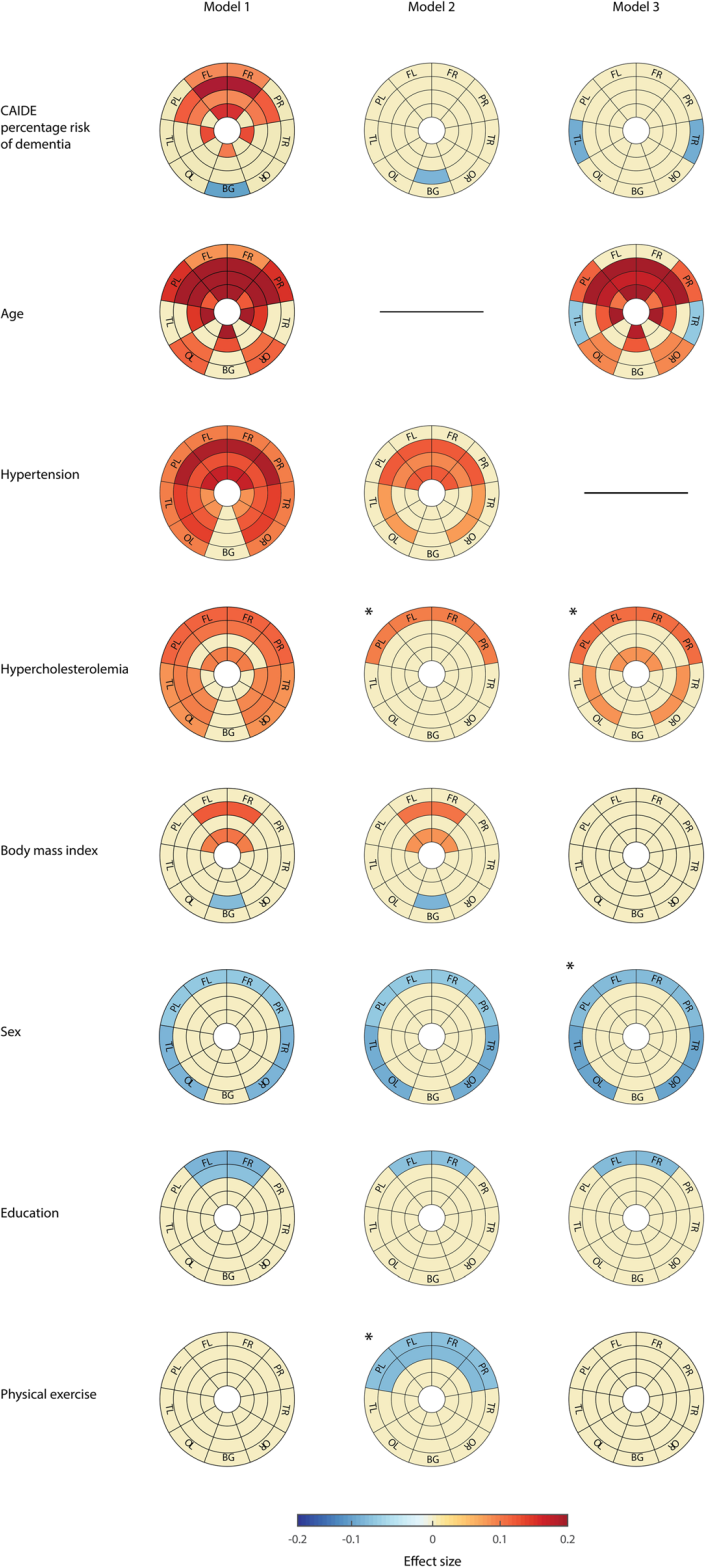
Regional patterns of WMH associations with CAIDE-I and each of the individual risk factors included in the scale. The individual risks of CAIDE-I are: age, hypertension, hypercholesterolemia, body mass index, sex, education, and physical exercise. Model 1 shows direct correlations without covariates (first column). Model 2 and Model 3 show correlations against WMH correcting by age and hypertension, respectively (second and third column). Effect sizes of the correlation are colored only on regions that showed significant association (*p* < 0.05 FDR-corrected). Hot colors represent positive correlations between WMH and each particular condition, and cold colors negative associations. In dichotomic comparisons, hypertensive, hypercholesterolemic, men, and active participants were set as reference groups. *Model of reference due to demographic characteristics of this group. BG basal ganglia, CAIDE-I Cardiovascular Risk Factors, Aging, and Incidence of Dementia percentage risk of dementia (model without APOE), FL left frontal lobe, FR right frontal lobe, PL left parietal lobe, PR right parietal lobe, OL left occipital lobe, OR right occipital lobe, TL left temporal lobe, TR right temporal lobe

CAIDE percentage risk of dementia was significantly associated with anterior WMH, specifically in all frontal lobe and parietal DWM (layers 2 and 3) in Model 1 (Fig. 2, first column). Periventricular WMH in the temporal lobe and basal ganglia also showed significant increments with higher CAIDE percentage risk of dementia.

Regarding individual factors, age and hypertension showed the highest effect sizes in line with the global analysis. They both showed a widespread association across almost all regions of the brain. The use of medication for controlling hypertension was the condition most highly correlated with WMH from those considered for assessing hypertension, with almost all regions having a significant and high effect (Additional file 1: Figure S3). Hypercholesterolemia also displayed significant widespread associations with WMH but with lower effect sizes. BMI showed significant associations with periventricular WMH in the frontal, parietal, and temporal lobes, as well as with DWM lesions in the frontal lobe. Consistent with the CAIDE analysis, we found a negative association between BMI and WMH in one of the external layers of the basal ganglia. Women displayed a juxtacortical pattern of higher WMH load in all lobes compared with men. The effect of education reached significance in the frontal external layers, with higher education being protective against WMH. Finally, physical exercise did not show any significant association with WMH.

The middle and right columns of Fig. 2 show the results after correcting for age (Model 2) and hypertension (Model 3) for CAIDE and individual risk factors. CAIDE percentage risk showed only a negative association with WMH in basal ganglia when corrected for age (Model 2). Negative associations, but now in the parietal lobe, remained when we accounted for hypertension status (Model 3). All these regions became nonsignificant if any secondary driver (BMI or hypercholesterolemia) was included in the model (data not shown).

Age and hypertension still showed significant regional WMH associations after correction by the other variable (Model 3 and Model 2, respectively). In Model 3, age was significantly correlated with WMH burden in almost all the same regions as with Model 1. However, a negative association appeared in the juxtacortical layer of the temporal lobe in this model, as in the CAIDE analysis. Internal and DWM layers of anterior areas showed a significant association with hypertension in Model 2 (age-corrected). Also, some DWM of temporal and occipital regions remained significantly associated.

BMI and hypercholesterolemia were highly correlated with hypertension in our sample (*p* < 0.001, Additional file 1: Figure S2 and Table S4) and, accordingly, the direct pattern of WMH (Model 1) disappeared (BMI) or was reduced (hypercholesterolemia) when adjusting by this factor (Model 3). Hypercholesterolemia was also highly correlated with age (Additional file 1: Table S4), and when we corrected for this factor only juxtacortical areas of the frontal and parietal lobes remained above the significance threshold.

Sex differences in juxtacortical areas remained after controlling for age or hypertension status (Models 2 and 3) which is relevant as, in our cohort, hypertension was less prevalent in women than in men (21.3% vs 33.8%; *p* < 0.001, Additional file 1: Table S5).

Higher levels of education and physical exercise were associated with lower regional WMH load in juxtacortical layers of the anterior areas. For education, the frontal areas remained significant in all three models. This is remarkable since age and education were negatively correlated in our cohort (Additional file 1: Figure S2). In the case of physical exercise, the only model that showed regional WMH burden associations was Model 2 (age-corrected). However, that was the best model for this variable as age was significantly different for participants not physically active vs physically active (56 vs 58 years old; *p* = 0.069, Additional file 1: Table S6).

**Fig. 3:**
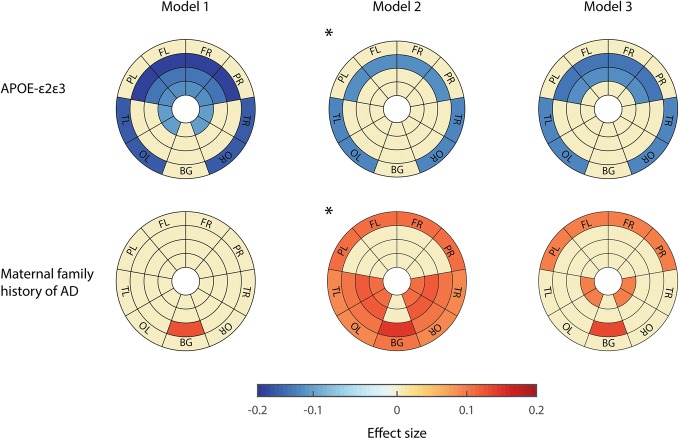
Regional patterns of WMH correlation with APOE and family history of AD. These models show correlations without any covariate (Model 1), correcting for age (Model 2), and correcting for hypertension (Model 3). Effect sizes of the correlation are shown only on regions that presented significant association (*p* < 0.05 FDR-corrected). Hot colors represent positive correlations between WMH and each particular condition, and cold colors negative associations. APOE-ε3ε3 carriers and participants without family history of AD were set as reference group. *Model of reference due to demographic characteristics of this group. AD Alzheimer’s disease, APOE apolipoprotein E, BG basal ganglia, FL left frontal lobe, FR right frontal lobe, PL left parietal lobe, PR right parietal lobe, OL left occipital lobe, OR right occipital lobe, TL left temporal lobe, TR right temporal lobe

Figure 3 shows the regional analysis with hereditary AD risk factors. *APOE* group differences were only significant in *APOE-ε2* carriers. They showed a protective effect against WMH in external white matter layers of all lobes. Differences were more pronounced in Model 1 because *APOE-ε2* carriers were significantly younger than *APOE-ε3/ε3* individuals in our cohort (56 vs 60 years old; *p* < 0.001, Additional file 1: Table S7). Therefore, Model 2 that corrected for the between-group difference in age should be regarded as the model of reference. It has to be noted that *APOE-ε2* carriers also differed to *APOE-ε3/ε3* individuals in the proportion of participants with hypercholesterolemia (15.5% vs 32.9%; *p* = 0.001, Additional file 1: Table S7).

Finally, we only found regional differences in WMH load in those subjects with a maternal family history of AD against those without. In these subjects, increased WMH burden was detected mainly in the temporal and occipital areas, but also in the juxtacortical frontal, parietal, and basal ganglia areas. The group of participants with a maternal family history of AD were younger than the group without a family history in our cohort (55 vs 60 years old; *p* < 0.001, Additional file 1: Table S8). Therefore, for this comparison, Model 2 should be regarded as the reference since it accounted for the between-group differences in age.

## Discussion

### Global analysis

In this study, we sought to extend previous findings reporting associations between AD risk factors against global and regional patterns of WMH in a relatively younger and cognitively unimpaired sample. Even though our middle-aged/late middle-aged participants displayed very low WMH burden compared with previously studied clinical cohorts [27, 33, 36] and a low dementia risk, a significant association was found between CAIDE and global WMH load. Higher WMH burden with increased CAIDE dementia risk scores are in line with previous literature in late-life [30, 31] and in late-midlife participants with mild cognitive impairment and subjective cognitive decline [51]. The fact that similar results were obtained in our younger sample of cognitively unimpaired participants suggests that increased WMH load might be an early mechanism through which cardiovascular factors increase the risk of dementia [12, 52, 53]. This effect did not survive correction by age, thus suggesting that this association is either directly mediated by age or by age-related factors.

As expected, there was an association between hypertension and global WMH load that survived correction by age. This result suggests that tight control of systolic hypertension in midlife might be an effective strategy to prevent late-life cognitive decline. Recently published results from a randomized clinical trial (SPRINT-MIND) [54] in individuals with increased cardiovascular risk, but without diabetes, showed that control of systolic blood pressure below 120 mmHg resulted in 19% fewer cases of mild cognitive decline over 3 years [55]. Indeed, additional evidence supports that the effect of cardiovascular disease on brain health is stronger in midlife [56, 57].

### Regional analysis

Our regional analysis of WMH distribution also showed interesting results. We observed that the CAIDE percentage risk of dementia was significantly correlated with WMH load in frontal and deep parietal areas. DWM in these territories is irrigated by the distal branches of superficial perforating arterioles of the anterior cerebral artery and the superior division of the middle cerebral artery. Our result suggests that early small vessel disease, specifically in these territories, might prompt future cognitive decline in a particularly severe way. Actually, previous reports have shown that parietal WMH burden is associated with increased risk of AD [58], whereas frontal WMH is associated with nonspecific cognitive impairment [36, 59]. It is unclear, however, whether this regional pattern is linked to mechanisms related to the distinct features of the arteriolar wall and/or perivascular space in deep white matter, or just reflect increased susceptibility of these areas to small vessel disease at the earliest stages [60].

Regarding individual AD risk factors, age and hypertension displayed the strongest and most widespread association with WMH load, consistent with previous reports [33, 61], especially in the anterior regions and in line with a recent study [62]. The comparison of the results before and after accounting for the effect of these two main drivers of WMH burden allowed us to reveal specific patterns of WMH load in association with the other risk factors. Both risk factors showed significant regional associations with WMH burden once corrected for the other. This result illustrates that even though they were highly correlated (Additional file 1: Figure S2), their association with WMH load seems to be independent, at least regionally.

We also found interesting results when we looked at the association of regional WMH burden and the three criteria used to assess hypertension. Records of antihypertensive medication were more strongly associated with this regional pattern of WMH load than self-reported clinical history of hypertension or a systolic blood pressure over 140 mmHg (Additional file 1: Figure S3). This result supports the criteria selected here to define hypertension for the study of its impact on WMH burden.

The frontal lobe was particularly sensitive to factors associated with healthy lifestyle habits, such as BMI, hypercholesterolemia, education, or physical exercise. Education and physical exercise showed a protective effect on frontal WMH load, as previously reported [33], whereas BMI and hypercholesterolemia had a negative impact on WMH burden. However, all these factors apart from physical exercise were highly correlated with hypertension in our sample (*p* < 0.001, Additional file 1: Figure S2), and the pattern of the WMH effect disappeared when adjusting for this factor (Model 3).

Regarding nonmodifiable factors other than age, female sex was associated with higher juxtacortical WMH load than male sex. A greater WMH burden in women may seem counterintuitive given that men tend to have a worse vascular risk factor profile (as actually occurred in our sample: 21.3% hypertensive women vs 33.8% men; *p* = 0.001, Additional file 1: Table S5). Nevertheless, this result survived correction for hypertension and is in agreement with previous studies [1, 63, 64] showing females to have more lesions than men, especially across the external layers of white matter [33] that persists even after adjustment for midlife vascular risk factors [63]. Some of the possible explanations previously proposed for these results were a higher prevalence of arterial stiffness in women than in men and sexual differences in white matter microstructure [63].

Regarding *APOE*, we found the *ε2* allele to be protective against WMH. This effect was present in all brain lobes either in DWM or juxtacortical areas. It could be speculated that the *APOE-ε2* protective effect is exerted through the protective effect of this allele against hypercholesterolemia. Consistent with this hypothesis, the *APOE-ε2* group in our cohort had a significant lower proportion of hypercholesterolemic participants (15.5% vs 32.9%; *p* = 0.001, Additional file 1: Table S6) and the regional pattern of WMH associations with these two factors was very similar (see the first column of Figs. 2 and 3).

Although we expected a higher prevalence of pathological WMH burden for *APOE-ε4* homozygotes looking at previous studies using the same sample [17], we only found a statistically non-significant increase in WMH load in this group. A possible explanation reconciling the nonsignificantly increased WMH load in this volumetric study and our previous finding of higher pathological levels of WMH in *APOE-ε4* homozygotes may lie in a faster rate of WMH progression, thus reaching pathological levels earlier, compared with *ε4* heterozygotes and noncarriers as previously reported in longitudinal studies [20, 23]. To confirm this possibility, longitudinal follow-up of this cohort will ensue.

When correcting for the significant between-group age differences (Model 2), we also detected a higher global and regional WMH load in participants with a maternal history of AD than in those with no familiar history. These regional associations were mainly found in the temporal and occipital lobes but were also present in other juxtacortical areas. This finding seems to differ from a previous report that did not observe any association between family history and WMH burden in a comparable cohort [27], even though the family history classification in this previous study was performed irrespective of the age of onset of AD. Our finding of increased WMH burden in regions that particularly increase the risk of AD [36] may contribute to the observed higher AD prevalence in individuals with a maternal family history compared with those with a paternal one [65].

We would like to highlight that in another study performed in the same ALFA cohort we found significant effects of global and regional WMH on cognition [35]. These findings are especially important as they point out that WMH impacts on cognition even in cognitively normal participants, presumably by the effect of AD risk factors. More importantly, the fact that we found a correlation between higher WMH burden in the frontal lobe and lowered executive function and memory, and that WMH load for this same reason was impacted by modifiable risk factors, allows us to hypothesize that controlling modifiable AD risk factors can impact cognition mediated by WMH burden. However, a more exhaustive study should be performed to better understand these links.

This study has some strengths and limitations that should be noted. One of the strengths is the composition of our cohort, formed by middle-aged/late middle-aged cognitively healthy participants enriched for a family history of AD. This composition allowed us to study WMH burden without the confounding effect of the presence of other comorbidities. In addition, the sample contained a relatively large number of *APOE-ε4* and *APOE-ε2* carriers, which allowed us to look for differences in WMH load as a function of genotype. Nevertheless, the characteristics of our cohort also caused some difficulties that resulted in several limitations. The high percentage of relatively young and WMH-free individuals resulted in a severely skewed distribution of WMH volumes, which prevented us from the use of parametric statistics. This fact limited the statistical power of our analysis and complicated the removal of confounding effects and the assessment of interactions between different factors. Nevertheless, this unavoidable limitation is inevitably shared by similar studies. However, our methodological approach allowed us to detect significant associations without needing to dichotomize either CAIDE or WMH load values, unlike previous studies. In addition, we used additional statistical models accounting for the two main drivers of WMH load: age and hypertension. This procedure allowed us to disentangle their global effect from that of other risk factors that displayed distinct local associations with WMH load. In addition, when analyzing other factors such as sex, *APOE* status, or family history, these complementary models allowed us to correct for between-group demographic imbalances in these two main WMH drivers. Some previous articles presented analyses correcting for overall lesion load as an aggregated proxy of all WMH risk factors, irrespective of whether they were measured or not. However, this procedure may lead to results more difficult to interpret (such as an apparently negative correlation between WMH load and age in brain regions actually not developing any lesions). On the other hand, our approach of correcting for the two main WMH drivers reports comparable benefits while keeping results more directly interpretable and allowing the correction of imbalances in post-hoc group comparisons. Another obvious limitation is the cross-sectional nature of our study, which prevents us from assessing the impact of WMH load and its longitudinal change on the clinical progression of the studied individuals. Finally, the lack of participant amyloid status is also a limitation of the study that is currently being addressed.

## Conclusions

Taking our results together, we have characterized the cerebral patterns of WMH load as a function of dementia risk factors in a cohort of middle-aged/late middle-aged cognitively unimpaired individuals. We found significant correlations between global and regional patterns of WMH load vs CAIDE percentage risk of dementia as well as with individual risk factors. Age and hypertension were the main drivers of the association between WMH and CAIDE, and were associated with a widespread regional effect pattern. Modifiable risk factors such as BMI and hypercholesterolemia were also associated with global and regional WMH, though to a lower degree. Unmodifiable AD-related factors such as sex, *APOE-ε2*, and maternal family history were associated with distinct regional patterns of WMH that persisted after adjustment for age and hypertension status. Our results suggest that even small and localized levels of WMH load may increase the risk of late-life dementia. These findings highlight the important effect of modifiable and nonmodifiable risk factors on WMH even when the burden of WMH is low. Due to their lack of other comorbidities and the young age in our cohort, the participants in our study represent a target population for the control of modifiable risk factors to avoid the development of WMH and to prevent or delay the onset of cognitive decline.

## Additional file

Additional file 1: **Figure S1.** Description of global and regional WMH burden. **Figure S2.** Cross-correlation between CAIDE-I percentage of dementia and its individual risk factors. **Figure S3.** Regional patterns of WMH associations with hypertension measured by different classifications. **Table S1.** Risk factors taken into account to derive CAIDE dementia risk scores and their corresponding points assigned. **Table S2.** Associations between global WMH and individual conditions to assess hypertension. **Table S3.** Comparison of CAIDE risk factors between hypertensive and nonhypertensive participants. **Table S4.** Comparison of CAIDE risk factors between nonhypercholesterolemic and hypercholesterolemic participants. **Table S5.** Comparison of CAIDE risk factors between women and men. **Table S6.** Comparison of CAIDE risk factors between physically inactive and active participants. **Table S7.** Comparison of CAIDE risk factors between *APOE-ε2* carriers and *APOE-ε3* homozygotes. **Table S8.** Comparison of CAIDE risk factors between participants with maternal family history and no family history of AD. (DOCX 1802 kb)

## Abbreviations

AD: Alzheimer’s disease
APOE: Apolipoprotein E
BMI: Body mass index
CAIDE: Cardiovascular Risk Factors, Aging, and Incidence of Dementia
CDR: Clinical Dementia Rating
CI: Confidence interval
DWM: Deep white matter
FDR: False discovery ratio
FLAIR: Fluid attenuation inversion recovery
GIF: Geodesic Information Flows
GRE: Gradient echo
MIS: Memory Impairment Screen
MMSE: Mini-Mental State Examination
MRI: Magnetic resonance imaging
SF: Semantic fluency
TIV: Total intracranial volume
TO-BTII: Time-Orientation subtest of the Barcelona Test II
WMH: White matter hyperintensities
