# Supplementary material for: Spatial patterns of white matter hyperintensities associated with Alzheimer’s disease risk factors in a cognitively healthy middle-aged cohort
Source: Alzheimers Res Ther. 2019 Jan 24;11:12. doi: 10.1186/s13195-018-0460-1 (PMC6346579; doi:10.1186/s13195-018-0460-1)
Supplement: Supplementary file 1 — Figure S1. Description of global and regional WMH burden. Figure S2. Cross-correlation between CAIDE-I percentage of dementia and its individual risk factors. Figure S3. Regional patterns of WMH associations with hypertension measured by different classifications. Table S1. Risk factors taken into account to derive CAIDE dementia risk scores and their corresponding points assigned. Table S2. Associations between global WMH and individual conditions to assess hypertension. Table S3. Comparison of CAIDE risk factors between hypertensive and nonhypertensive participants. Table S4. Comparison of CAIDE risk factors between nonhypercholesterolemic and hypercholesterolemic participants. Table S5. Comparison of CAIDE risk factors between women and men. Table S6. Comparison of CAIDE risk factors between physically inactive and active participants. Table S7. Comparison of CAIDE risk factors between APOE-ε2 carriers and APOE-ε3 homozygotes. Table S8. Comparison of CAIDE risk factors between participants with maternal family history and no family history of AD. (DOCX 1802 kb) [file 13195_2018_460_MOESM1_ESM.docx]

**Additional file 1**

*Spatial Patterns of White Matter Hyperintensities Associated to Alzheimer’s Disease Risk Factors in a Cognitively Healthy Middle-Aged Cohort*

Gemma Salvadó, Anna Brugulat-Serrat, Carole H. Sudre, Oriol Grau-Rivera, Marc Suárez-Calvet, Carles Falcon, Karine Fauria, M. Jorge Cardoso, Frederik Barkhof, José Luis Molinuevo, Juan Domingo Gispert, for the ALFA Study†


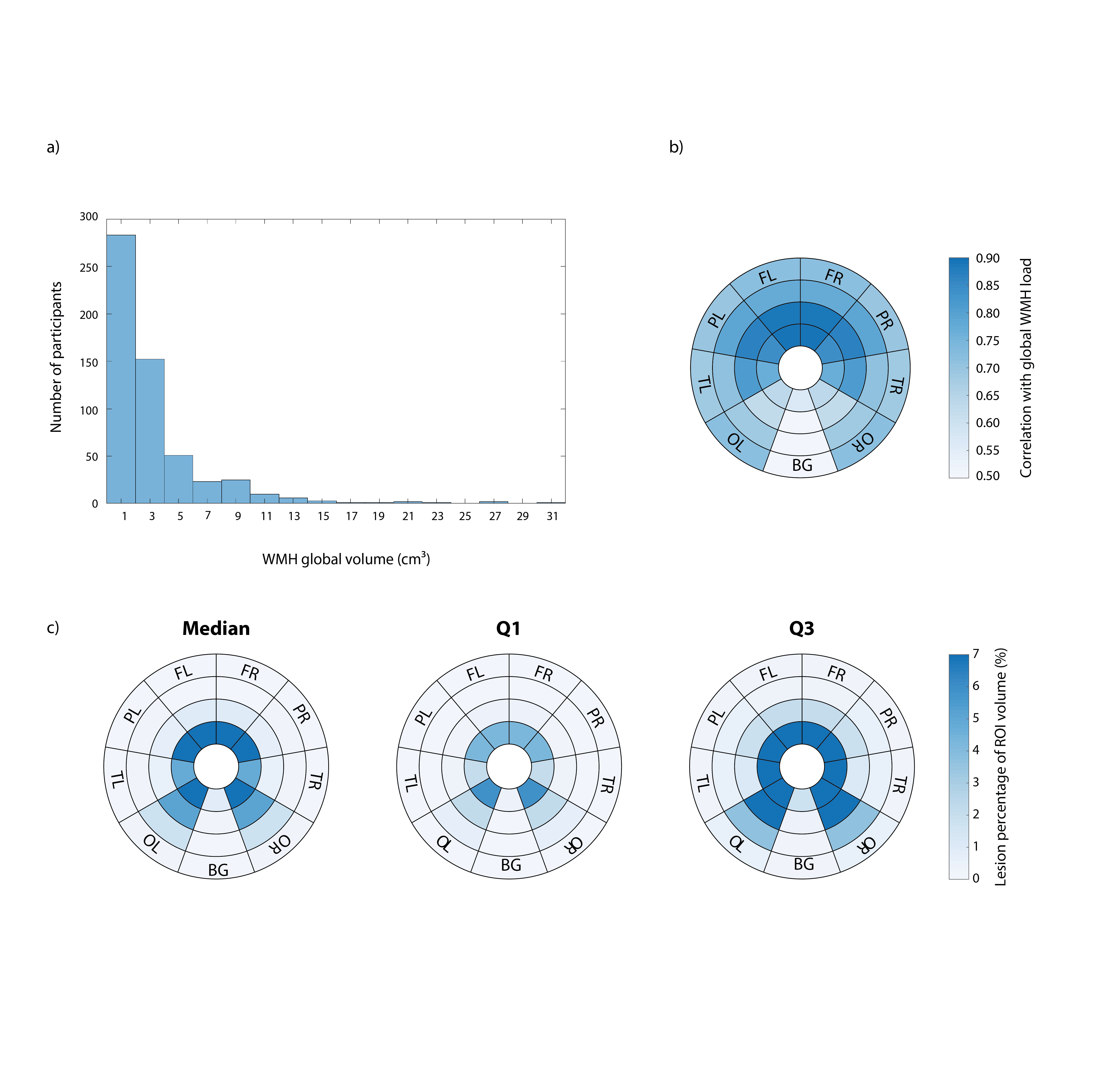


**Figure S1.** Description of global and regional WMH burden. a) Histogram of WMH load (n=561). b) Correlation between global and regional WMH burden. c) WMH percentage of ROI volume in each region (left: median, center: Q1 and, right: Q3).

Abbreviations: WMH: white matter hyperintensities, FR: frontal right, FL: frontal left, TR: temporal right, TL: temporal left, PR: parietal right, PL: parietal left, OR: occipital right, OL: occipital left, BG: basal ganglia, Q1: first interquartile and Q3: third interquartile.

**
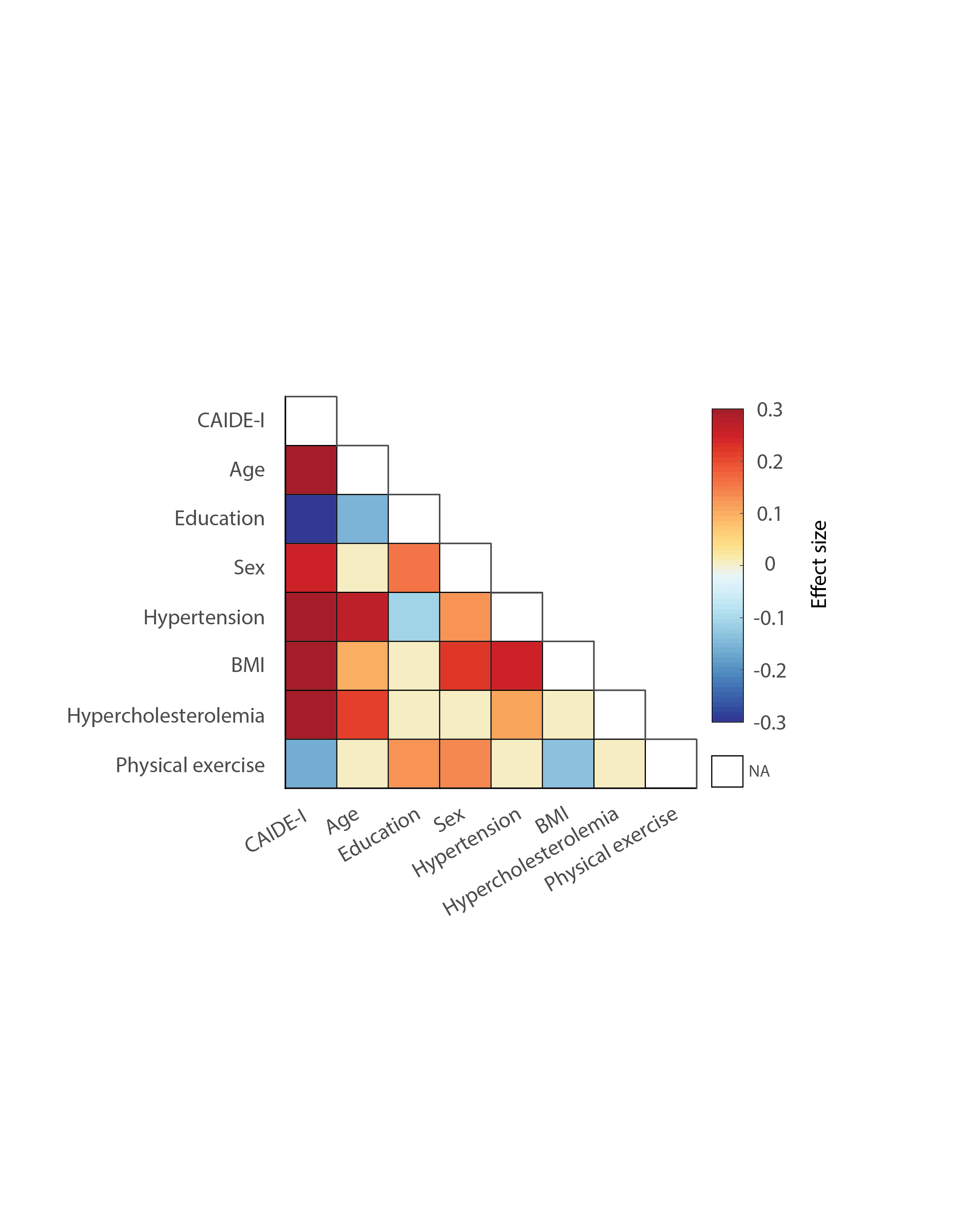
Figure S2.** Cross-correlation between CAIDE-I percentage of dementia and its individual risk factors. The colours indicate the effect size of the correlation. Warm colours indicate a positive correlation between factors and cold colours indicate a negative correlation (men and, hypertensive and hypercholesterolemic participants are the reference group for their categories). Squares that appear in beige showed no signifficant correlation (p>0.05), all the rest correlations were significant.


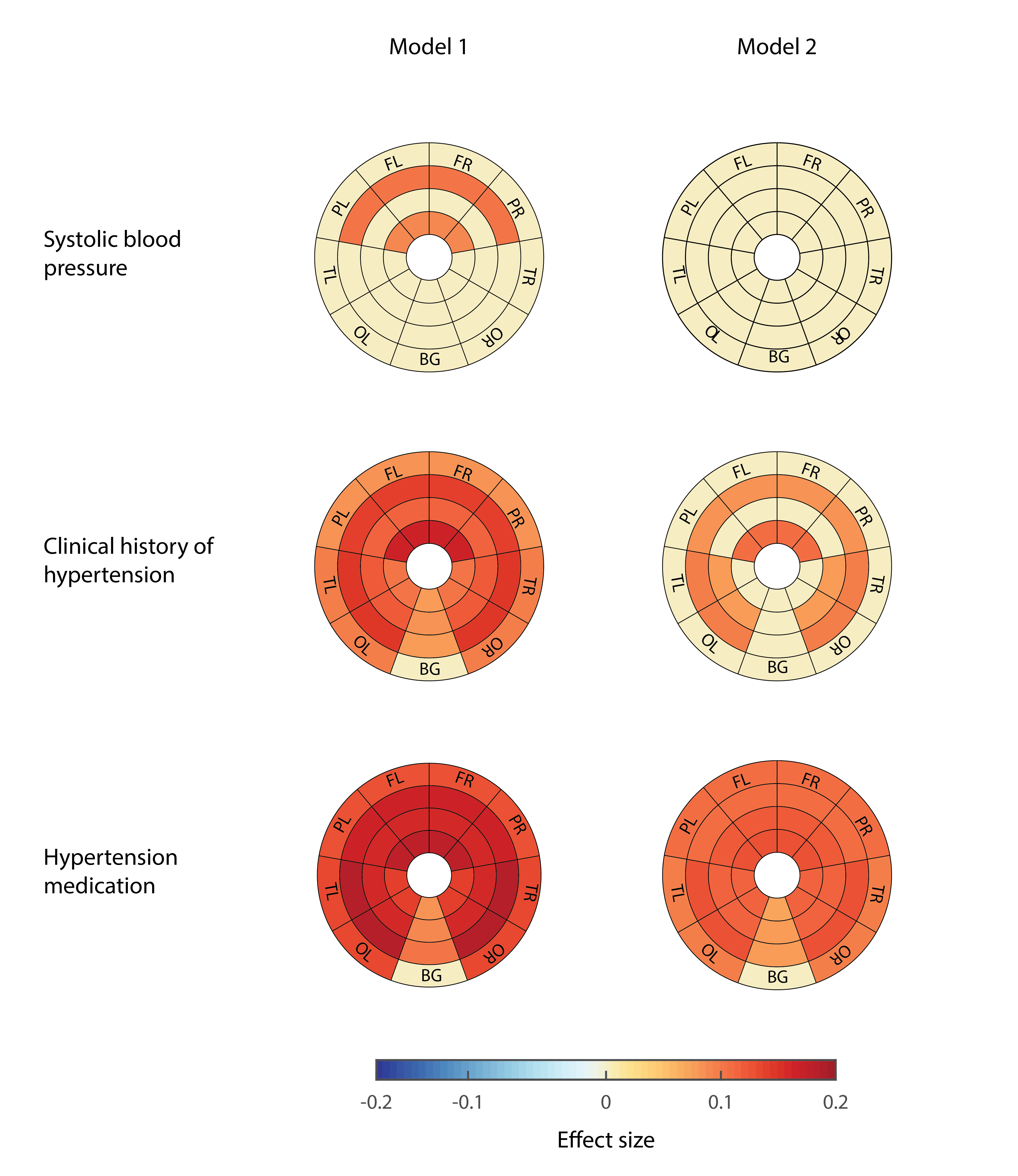


**Figure S3.** Regional patterns of WMH associations with hypertension measured assessed by different classifications. These classifications were: systolic blood pressure (hypertensive if systolic blood pressure above 140 mmHg), self-reported clinical history of hypertension or, use of hypertension medication. Model 1 shows direct correlations without covariates (first column) and Model 2 shows correlations against WMH covariating by age (second column). Effect sizes of the correlation are coloured only on regions that showed significant association (p<0.05 FDR-corrected). Hot corlours represent positive correlations between WMH and each particular condition, and cold colours negative associations.

Abbreviations: FR: Right frontal lobe, FL: left frontal lobe, TR: right temporal lobe, TL: left temporal lobe, PR: right parietal lobe, PL: left parietal lobe, OR: right occipital lobe OL: left occipital lobe, BG: Basal ganglia, WMH: white matter hyperintensities, FDR: false discovery ratio.

| Risk factor | CAIDE Dementia risk score |
| --- | --- |
| Age, years | |
| <47 | 0 |
| 47-53 | 3 |
| >53 | 4 |
| Education, years | |
| ≥10 | 0 |
| 7-9 | 2 |
| 0-6 | 3 |
| Sex | |
| Women | 0 |
| Men | 1 |
| Systolic blood pressure | |
| ≤140 mmHg | 0 |
| >140 mmHg | 2 |
| BMI, kg/m^2^ | |
| ≤30 | 0 |
| >30 | 2 |
| Hypercholesterolemia | |
| No | 0 |
| Yes | 2 |
| Physical activity | |
| Active | 0 |
| Inactive | 1 |
| TOTAL | 15 |

**Table S1:** Risk factors taken into account to derive CAIDE dementia risk scores and their corresponent points assigned.

|  | **Model 1** | | | **Model 2** | |
| --- | --- | --- | --- | --- | --- |
|  | Effect size  [95% CI] | p | Effect size  [95% CI] | | p |
| Systolic blood pressure | 0.05  [-0.03 - 0.14] | 0.103 | 0.03  [-0.06 - 0.11] | | 0.277 |
| Clinical history of hypertension | **0.13**  **[0.05 - 0.22]** | **<0.001** | **0.09**  **[0.00 - 0.18]** | | **0.014** |
| Medication for hypertension | **0.17**  **[0.09 - 0.26]** | **<0.001** | **0.13**  **[0.04 - 0.22]** | | **0.001** |

**Table S2:** Associations between global WMH and individual conditions to assess hypertension. These classifications were: by systolic blood pressure (hypertensive if systolic blood pressure above 140 mmHg), self-reported hypertension or, use of medication to control hypertension. Effect size [95CI%] and p-values are shown. The three models had as outcome variable WMH % of TIV. No covariates were used in Model 1 and age effect was corrected in Model 2. Significant results are shown in bold (p<0.05 FDR-corrected).

Abbreviations: WMH: white matter hyperintensities, CI: confidence interval, TIV: total intracranial volume, FDR: false discovery ratio.

|  | Non-hypertensive  (n=414) | Hypertensive  (n=147) | p |
| --- | --- | --- | --- |
| Age | **56 [47-69]** | **62 [49-73]** | **<0.001** |
| Hypecholesterolemia | **108 (26.1)** | **63 (42.9)** | **<0.001** |
| BMI | **25.9 [21.1-32.7]** | **28.0 [22.6-35.6]** | **<0.001** |
| Sex, women | **269 (65.0)** | **73 (49.7)** | **0.001** |
| Education | 15 [8-18] | 12 [8-18] | 0.241 |
| Physical activity | 272 (70.5) | 96 (73.8) | 0.461 |

**Table S3.** Comparison of CAIDE risk factors between hypertensive and non-hypertensive participants. These two groups are statistically different in age (p<0.001), hypercholesterolemia (p<0.001), BMI (p<0.001) and sex (p=0.001).

Abbreviations: BMI: body mass index.

|  | Non-hypercholesterolemic  (n=390) | Hypercholesterolemic  (n=171) | p |
| --- | --- | --- | --- |
| Age | **56 [47-70]** | **60 [48-71]** | **<0.001** |
| Hypertension | **84 (21.5)** | **63 (36.8)** | **<0.001** |
| BMI | 26.2 [21.2-33.6] | 26.9 [21.7-34.7] | 0.167 |
| Sex, women | 242 (62.1) | 100 (58.5) | 0.425 |
| Education | 15 [8-18] | 12 [8-18] | 0.087 |
| Physical activity | 250 (70.2) | 118 (73.8) | 0.413 |

**Table S4.** Comparison of CAIDE risk factors between non-hypercholesterolemic and hypercholesterolemic participants. These two groups are statistically different in age (p<0.001) and hypertension (p<0.001).

Abbreviations: BMI: body mass index.

|  | Men (n=219) | Women (n=342) | p |
| --- | --- | --- | --- |
| Age | 58 [47-71] | 57 [47-71] | 0.281 |
| Hypertension | **74 (33.8)** | **73 (21.3)** | **0.001** |
| Hypercholesterolemia | 71 (32.4) | 100 (29.2) | 0.425 |
| BMI | **27.5 [23.0-33.5]** | **25.7 [20.5-34.3]** | **<0.001** |
| Education | **15 [8-18]** | **12 [8-18]** | **<0.001** |
| Physical activity | **159 (79.1)** | **209 (66.3)** | **0.002** |

**Table S5.** Comparison of CAIDE risk factors between women and men. These two groups are statistically different in hypertension (p=0.001), BMI (p<0.001), education (p<0.001) and, physical activity (p=0.002).

Abbreviations: BMI: body mass index.

|  | Physically inactive (n=148) | Physically active (n=368) | p |
| --- | --- | --- | --- |
| Age | **56 [47-67]** | **58 [47-71]** | **0.069** |
| Hypertension | 34 (23.0) | 96 (26.1) | 0.461 |
| Hypercholesterolemia | 42 (28.4) | 118 (32.1) | 0.413 |
| BMI | **26.9 [21.3-35.4]** | **26.1 [21.2-32.7]** | **0.020** |
| Sex, women | **106 (71.6)** | **209 (56.8)** | **0.002** |
| Education | **12 [8-18]** | **15 [8-18]** | **0.004** |

**Table S6.** Comparison of CAIDE risk factors between physically inactive and active participants. These two groups are statistically different in age (p=0.069), BMI (p=0.020), sex (p=0.002) and, education (p=0.004).

Abbreviations: BMI: body mass index.

|  | *APOE-ε2ε3* (n=110) | *APOE-ε3ε3* (n=161) | p |
| --- | --- | --- | --- |
| Age | **56 [47-66]** | **60 [47-72]** | **<0.001** |
| Hypertension | 26 (23.6) | 47 (29.2) | 0.311 |
| Hypercholesterolemia | **17 (15.5)** | **53 (32.9)** | **0.001** |
| BMI | 26.2 [21.1-34.6] | 26.4 [21.8-33.7] | 0.847 |
| Sex, women | 67 (60.9) | 108 (67.1) | 0.297 |
| Education | **15 [8-18]** | **12 [8-18]** | **0.057** |
| Physical activity | 70 (68.0) | 107 (70.9) | 0.622 |

**Table S7.** Comparison of CAIDE risk factors between *APOE-ε2* carriers and *APOE-ε3* homozygotes. These two groups are statistically different in age (p<0.001), hypercholesterolemia (p=0.001) and, education (p=0.057).

Abbreviations: BMI: body mass index.

|  | No family history of AD (n=245) | Maternal family history of AD (n=190) | p |
| --- | --- | --- | --- |
| Age | **60 [48-72]** | **55 [47-69]** | **<0.001** |
| Hypertension | 67 (27.3) | 50 (26.3) | 0.810 |
| Hypercholesterolemia | 77 (31.4) | 58 (30.5) | 0.840 |
| BMI | 26.5 [21.4-33.3] | 26.1 [21.4-34.7] | 0.752 |
| Sex, Female | 155 (63.3) | 121 (63.7) | 0.928 |
| Education | 15 [8-18] | 12[8-18] | 0.195 |
| Physical activity | 166 (73.8) | 118 (65.9) | 0.086 |

**Table S8.** Comparison of CAIDE risk factors between participants with maternal and no family history of AD. These two groups are statistically different in age (p<0.001).

Abbreviations: BMI: body mass index.
